# Can China’s national comprehensive medical reform increase medical resources and reduce healthcare burden: evidence from Chinese provinces

**DOI:** 10.3389/fpubh.2024.1444840

**Published:** 2024-11-12

**Authors:** Xiangyu Fu, Xiang Ren, Qirui Chen

**Affiliations:** ^1^Department of Ophthalmology, West China Hospital, Sichuan University, Chengdu, China; ^2^Research Laboratory of Ophthalmology and Vision Sciences, State Key Laboratory of Biotherapy, West China Hospital, Sichuan University, Chengdu, China; ^3^School of Public Administration, Sichuan University, Chengdu, China

**Keywords:** national comprehensive medical reform (NCMR), medical resources, residents’ medical expenses, staggered difference-in-differences (DID), synthetic control method (SCM), provincial heterogeneity

## Abstract

**Objectives:**

China’s national comprehensive medical reform (NCMR) is an important attempt in the reform of healthcare system, and quantitative evaluation of its effect is of great significance for continuously deepening medical reform, grasping the reform direction, and building a healthy China. Therefore, focusing on medical resources and medical burden, this study aims to systematically explore the policy effectiveness and the provincial heterogeneity of NCMR, as well as the potential influencing factors.

**Methods:**

Utilizing the collected multi-period panel data of 31 provinces in mainland China in 2006–2021, we regarded the release of the two batches of pilot provinces in NCMR as a quasi-natural experiment and comprehensively adopted a Staggered difference-in-differences (DID) model and Synthetic Control Method (SCM), combined with word frequency statistics and grouping regression analysis.

**Results:**

NCMR can effectively increase the number of licensed physicians by 12.6% and reduce the *per capita* medical expense for in-patients by 7.2% in the pilot provinces. Furthermore, the NCMR policy effect in different pilot provinces shows various characteristics, and only Jiangsu, Zhejiang, and Chongqing achieve both the growth of medical resources and the reduction of medical expenditure. Meanwhile, word frequency statistics are conducted based on related policy descriptions and news reports on the official websites, so as to summarize the specific policy means in the three provinces, and provide a reference for other provinces to practice the healthcare reforms. Besides, extensibility analysis shows that the effect of NCMR is affected by the population aging and health status. Groups with low degree of population aging (low-AG)/high population health status (high-HE) performed a more obvious reform effect.

**Conclusion:**

This study provides beneficial policy implications for increasing medical resources, reducing medical burden, and promoting medical reform process.

## 1 Introduction

As a private economic product with significant social welfare implications, the healthcare system is highly complex, requiring a balance between equity and efficiency for its development (1). Additionally, the provision of health services involves multiple tasks, often leading to conflicts (2). Furthermore, information asymmetry and positive externalities are inherent challenges (3). Consequently, motivating healthcare system reform remains both a global phenomenon and a persistent worldwide challenge.

As the largest developing country, China has experienced an economic development miracle since its reform and opening-up, significantly improving the health of its citizens. However, the development of the healthcare system has lagged behind (4), with the supply of public medical resources struggling to meet the growing demand for high-quality healthcare services (5). In response, China initiated healthcare system reforms. These efforts began in the 1980s and entered a phase of adjustment and innovation in 2006. Unfortunately, these early reforms failed to effectively reduce the social burden of medical care and produced no significant improvements. In 2009, China launched a new round of reforms, known as the New Healthcare Reform, which prioritized equity and aimed to provide basic healthcare of reasonable quality with adequate risk protection for all citizens (6). The New Healthcare Reform made significant progress in three key areas: medical insurance, drug pricing, and public hospitals (7), and essentially established the framework for comprehensive healthcare system reform.

With the continued implementation of the New Healthcare Reform, the complexity and unique characteristics of the healthcare system became more evident. Conflicts between supply and demand, doctors and patients, and urban and rural areas intensified, and resource allocation remained inefficient (8). Consequently, issues related to the accessibility and affordability of medical services persisted, commonly referred to in Chinese as “kan-bing-nan” and “kan-bing-gui.”

In the Chinese context, “kan-bing-nan” refers to “difficulties in accessing medical treatment,” underscoring the challenge of healthcare accessibility. Specifically, the insufficient supply and uneven distribution of medical resources fail to meet the growing demand for services. Furthermore, the difficulty in implementing a primary-level first-diagnosis system contributes to the inefficient allocation of high-quality healthcare resources.

On the other hand, “kan-bing-gui” denotes the “high cost of medical treatment,” focusing on the issue of healthcare affordability. Due to restrictions on the pricing of diagnostic and treatment services, the practice of using pharmacy profits to subsidize medical services has become widespread. Additionally, the parallel, multi-track medical insurance system has fragmented the mutual aid capacity of insurance, while differing health insurance financing mechanisms have widened disparities in *per capita* funding.

To consolidate reform achievements and innovate new approaches, the Leading Group for Medical Reform of the State Council of the People’s Republic of China decided to pilot the National Comprehensive Medical Reform (NCMR). Two batches of pilot provinces were selected, the first in January 2015 and the second in May 2016. The first batch included Jiangsu, Anhui, Fujian, and Qinghai provinces, while the second consisted of Shanghai, Zhejiang, Hunan, Chongqing, Sichuan, Shaanxi, and Ningxia.

Specifically, to improve accessibility of medical resources and affordability of medical services, the NCMR implemented various reform measures and achieved significant results. On the one hand, regarding improved accessibility, the pilot provinces continued to optimize the allocation of medical resources and established a system of tiered diagnosis and treatment (9). Key initiatives, such as the signing of family doctors, the provision of telemedicine services, and the development of an integrated healthcare system, facilitated the distribution of high-quality medical resources to lower levels, allowing common and chronic diseases to be treated promptly in primary healthcare institutions. Additionally, online services like appointment booking, test result inquiries, and mobile payment options have greatly enhanced the convenience of seeking medical care for residents.

On the other hand, to reduce medical expenses, the NCMR used drug reform as a critical entry point, lowering inflated drug prices through centralized bidding and procurement processes. Moreover, Medicare coverage was expanded, while personal out-of-pocket expenses were reduced (10). In some pilot provinces, the role of Medicare security was further strengthened by increasing the reimbursement rate for hospitalization costs for serious illnesses among the poor to 90%.

Overall, the NCMR focused on addressing the issues of “kan-bing-nan” and “kan-bing-gui,” and successfully implemented a series of reform measures in 11 pilot provinces, all aimed at resolving healthcare challenges. But what are the real-world effects of the NCMR? Has it effectively improved access to medical resources and the affordability of medical services? Are there differences in the policy impacts and specific strategies among the various pilot provinces? Are there other influencing factors at play?

**Figure 1:**
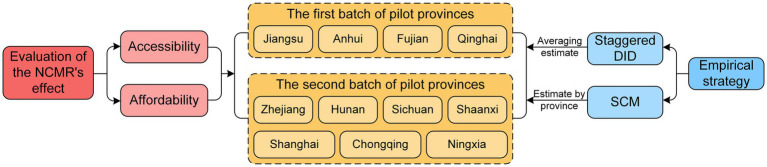
Research perspective and empirical strategies. NCMR, national comprehensive medical reform; DID, difference-in-differences; SCM, synthetic control method.

To address these questions, this paper treats the introduction of the two batches of pilot provinces under the NCMR as a quasi-natural experiment and employs a staggered difference-in-differences (DID) model to empirically analyze the average policy effect of NCMR on accessibility and affordability. Additionally, the Synthetic Control Method (SCM) is used to evaluate the policy effectiveness in each pilot province individually and to explore the heterogeneity between provinces. Furthermore, the impact of population aging and regional health status on policy effectiveness is also investigated. The detailed research perspectives and empirical strategies are illustrated in Figure 1.

The marginal contribution of this study lies in two key areas. First, it focuses on the policy implications of the NCMR by addressing the practical issues of “kan-bing-nan” and “kan-bing-gui.” By integrating the accessibility of medical resources with the affordability of medical services, we provide a comprehensive and systematic evaluation of the NCMR’s policy effects. Second, using multi-period panel data from 31 provinces in mainland China (excluding Hong Kong, Macao, and Taiwan) from 2006 to 2021, we employ both the Staggered DID model and the SCM to scientifically assess the overall policy impact of the NCMR. This approach also allows for province-specific evaluations, exploring provincial heterogeneity and other influencing factors. These contributions aim to help policymakers better understand the successes and limitations of healthcare reform.

## 2 Literature review

Addressing the availability of medical resources and the affordability of medical services is not only a key objective of healthcare reform but also an effective means of improving public well-being. As a result, many studies have focused on the factors influencing medical resource supply and residents’ medical expenditures.

In terms of resource supply, a strong correlation has been observed between regional economic development and the abundance of medical resources (11). Similarly, factors such as government health expenditure, population size, and the number of medical schools play significant roles in determining the distribution of high-quality medical resources (12). Lu et al. (13) examined the impact of referral reforms on resource accessibility, concluding that while the referral system improved access to public hospitals, it also exacerbated healthcare resource disparities between townships and urban districts.

Regarding residents’ medical expenditures, environmental degradation has been shown to increase public health spending, subsequently raising individual medical costs (14). Additionally, pollution-intensive industrial clusters have been found to increase urban residents’ healthcare expenses (15). Conversely, the large-scale development of digital infrastructure can enhance healthcare efficiency, thereby reducing individual medical burdens and helping to narrow wealth disparities (16).

In evaluating healthcare system reforms, much of the existing literature has focused on their impact on the demand for medical resources and the supply of healthcare services, often reflected in residents’ medical expenditures and the income structure of hospitals. Baji et al. (17) used Kakwani indexes to assess the progressivity of household healthcare expenditures in Hungary before, during, and after healthcare reforms, finding that informal payments became less regressive during the reform period. They concluded that equity in healthcare financing could be improved by eliminating informal payments while increasing co-payments (17).

Similarly, Limwattananon et al. (18) evaluated the effects of Thailand’s healthcare reforms on out-of-pocket healthcare expenditures using a DID model, revealing an average reduction of 28% in out-of-pocket spending, accompanied by increased utilization of inpatient and outpatient services. Zhu et al. (19) also employed the DID method to show that while drug expenditures decreased in Chinese public hospitals, spending on medical care increased due to price changes. Liu et al. (20) noted that two rounds of comprehensive public hospital reforms in Beijing contributed to lowering direct medical payments for in-patients with coronary heart disease.

In terms of hospital income structure, data from Beijing’s public hospital reforms showed that the proportion of revenue from drug sales significantly declined, while some outpatients were successfully transferred from tertiary hospitals to primary healthcare institutions, such as community health centers. However, the use of CT and MRI increased unexpectedly (21). Another study demonstrated a reduction in the proportion of revenue from drug sales and medical consumables, alongside a rise in revenue from medical consulting services. This research concluded that the comprehensive public hospital reforms effectively curbed medical expenditures and optimized hospital income structures (22).

The above analyses show that while previous studies have thoroughly examined healthcare reform policies and their impacts across various countries and regions, the NCMR in China has not been fully explored. Most related research has focused on specific provinces or cities, using limited evaluation metrics and lacking a systematic, multi-dimensional assessment across all pilot provinces. Additionally, most studies relied on average effect estimations, with little investigation into the provincial heterogeneity of policy outcomes.

To address these gaps, this paper employs both the Staggered DID model and the SCM to evaluate the NCMR from two key dimensions: resource accessibility and price affordability. It also explores influencing factors, providing a valuable addition to the existing literature and offering a more comprehensive and accurate understanding of the policy’s strengths and weaknesses.

## 3 Materials and methods

### 3.1 Variables and data sources

Addressing the difficulties of “kan-bing-nan” and “kan-bing-gui” is the primary objective of China’s NCMR. Therefore, we evaluated the policy’s effectiveness from two key dimensions: the accessibility of medical resources and the affordability of medical services.

Based on data availability and relevant studies (23, 24), we selected three indicators to measure accessibility: the number of licensed physicians (*Doctor*), registered nurses (*Nurse*), and sickbeds in medical institutions (*Sickbed*) per 1,000 population. For evaluating the affordability of medical services, we chose two indicators representing residents’ medical costs: *per capita* medical expenses for outpatients (*Out-patient*) and *per capita* medical expenses for inpatients (*In-patient*) (25).

To account for inflation, we deflated the expense-related indicators using the consumer price index, with the initial year of the data serving as the base period. All five indicators were transformed using logarithmic scales.

Additionally, the accessibility of medical resources and the affordability of health services in a region may be influenced by whether the region is an NCMR pilot province, as well as by various regional characteristics or random factors. Therefore, we included a series of control variables from different perspectives: regional environment (26, 27), population (12), economy (11), and industrial structure (28). The selected control variables were: air pollution (*AP*), represented by sulfur dioxide emissions; water pollution (*WP*), represented by chemical oxygen demand emissions; environmental greening (*EG*), expressed as *per capita* park green area; population size (*PS*), expressed as total population; economic development (*ED*), quantified by *per capita* GDP; and industrial structure (*IS*), quantified by the proportion of added value of the secondary industry in GDP.

Non-proportional data were logarithmically transformed and winsorized at the 1st and 99th percentiles before regression analysis. This process helps mitigate the influence of extreme values on the estimates without reducing the sample size.

This study focuses on 31 provinces in mainland China (excluding Hong Kong, Macao, and Taiwan) over the period from 2006 to 2021. Data for all indicators were obtained from the China *Health and Family Planning Statistical Yearbook, the China Health Statistics Yearbook, the China Statistics Yearbook on Environment*, and the National Bureau of Statistics of China website. Missing values were addressed using interpolation. Descriptive statistics for the main variables are presented in Table 1.

**Table 1:** Descriptive statistics.

Variables	Observations	Mean	SD	Min	Max
*Doctor*	496	1.884	0.707	0.748	5.500
*Nurse*	496	2.250	0.978	0.560	6.360
*Sickbed*	496	4.726	1.481	1.600	8.340
*Out-patient*	496	4.965	0.340	3.615	6.161
*In-patient*	496	8.582	0.368	7.678	9.833
*AP*	496	12.476	1.500	7.550	14.341
*WP*	496	12.722	1.066	9.776	14.405
*EG*	496	11.922	3.155	5.890	19.960
*PS*	496	8.112	0.848	5.704	9.404
*ED*	496	10.549	0.615	9.151	11.963
*IS*	496	41.964	8.356	17.254	59.388

*Doctor*, the number of licensed physicians per 1,000 population; *Nurse*, the number of registered nurses per 1,000 population; *Sickbed*, the number of sickbeds in medical institutions per 1,000 population; *Out-patient*, per capita per time medical expense for out-patients; *In-patient*, per capita medical expense for in-patients; *AP*, air pollution; *WP*, water pollution; *EG*, environmental greening; *PS*, population size; *ED*, economic development; *IS*, industrial structure; SD, standard deviation.

### 3.2 Empirical strategies and model construction

#### 3.2.1 Staggered DID model

Existing studies commonly use the DID model to assess policy effects. This model combines “differences before and after the intervention” with “differences between the experimental and control groups” using panel data, effectively controlling for factors unrelated to the intervention and providing a true assessment of the policy’s impact.

In our study, we applied the DID model to evaluate the impact of China’s NCMR. Given that the pilot provinces were introduced at two different time points, we used the Staggered DID method as our benchmark model for estimating the average policy effect. This method is well-suited for assessing the effects of a policy implemented incrementally over time, aligning with our study design.

Based on the methodology of Beck et al. (29), the benchmark regression model used in the study is specified as follows:


(1) $$Y_{\mathrm{it}}=\alpha +\beta \times {\mathrm{NCMR}}_{\mathrm{it}}+\gamma X_{\mathrm{it}}+\delta_{t}+\mu_{i}+\varepsilon_{\mathrm{it}}$$

where *i* and *t* represent the province and year, respectively. $Y_{it}$ is the dependent variables, i.e., *Doctor*, *Nurse*, *Sickbed*, *Out-patient*, and *In-patient*, which represent the medical resources and expenditures for province *i* in year *t*. $NCMR_{it}$ indicates whether province *i* is the pilot province of NCMR in year *t* (1 for inclusion and 0 for non-inclusion). $X_{it}$ represents a series of control variables. $\delta_{t}$ is the time fixed effect $.\mu_{i}$ is the regional fixed effect. $\varepsilon_{\mathrm{it}}$ is the random error (Equation 1). We mainly focused on the regression coefficient β of $NCMR_{it}$ , which indicates the effect of NCMR.

#### 3.2.2 SCM

The SCM, first proposed by Abadie and Gardeazabal (30), aims to create a “counterfactual” comparison unit for policy-affected provinces. It does so by constructing a synthetic control group through weighted combinations of potential control units, effectively simulating the conditions of the pilot provinces had they not been affected by the NCMR. SCM offers several advantages: it reduces selection bias, addresses endogeneity, and mitigates subjectivity in the selection of the control group (31).

The application of SCM is as follows: The dependent variables, including *Doctor*, *Nurse*, *Sickbed*, *Out-patient,* and *In-patient*, in period t∈[1,T] of K+1 provinces are obtained. $S_{it}^{N}$ denotes the dependent variables of province *i* in year *t* when NCMR was not implemented, whereas $S_{it}^{I}$ denotes the variables when NCMR was implemented. If $T_{0}$ is the implementation node of NCMR and $t\leq T_{0}$ , $S_{it}^{N}=S_{it}^{I}$ , whereas when $t>T_{0}$ , $\alpha_{it}=S_{it}^{I}-S_{it}^{N}$ is the policy effect. For the pilot provinces, $S_{it}^{I}$ , but not $S_{it}^{N}$ , can be observed and was estimated using the model proposed by Abadie et al. (32):


(2) $$S_{it}^{N}=\delta_{t}+\theta_{t}Z_{i}+\lambda_{t}\mu_{i}+\varepsilon_{\mathrm{it}}$$

where $\delta_{t}$ is the time fixed effect. $Z_{i}$ indicates the predictive control variables, including *AP*, *WP*, *EG*, *PS*, *ED*, *ID*, and the dependent variables in 2007, 2009, and 2011. $\lambda_{t}$ is the common factor vector. $\mu_{i}$ represents the regional fixed effect. $\varepsilon_{it}$ is the unobservable instantaneous shock at the province level (Equation 2).

Consider the scenarios where the first province ( i=1 ) has implemented NCMR, whereas the remaining K provinces ( i=2,3,⋯,K+1 ) have not implemented it. In this scenario, $W=\left(w_{2},w_{3},\cdots ,w_{K+1}\right)$ is a weight vector, where $w_{k}\geq 0$ , $w_{2}+w_{3}+\cdots +w_{K+1}=1$ . The vector W represents a virtual synthetic control combination.

Abadie et al. (32) proved that when $t>T_{0}$ , $\overset{K+1}{\underset{k=2}{\sum }}w_{k}^{*}S_{kt}^{N}$ can be considered an unbiased estimate of $S_{1t}^{N}$ . The policy effect can be estimated as follows:


(3) $${\hat{\alpha }}_{1t}=S_{1t}^{I}-S_{1t}^{N}=S_{1t}^{I}-\overset{K+1}{\underset{k=2}{\sum }}w_{k}^{*}S_{\mathrm{kt}}^{N},t\in \left[T_{0}+1,\cdots \mathrm{,T}\right]$$

Using the aforementioned formulae, ${\hat{\alpha }}_{1t}$ (the policy effect) was estimated based on the weight vector $W^{*}$ (Equation 3).

## 4 Results

### 4.1 Results of staggered DID test

#### 4.1.1 Benchmark regression results

The average impact of NCMR on medical resources and expenditures in the pilot provinces was estimated using the Staggered DID model, as shown in Table 2. The first three columns represent the effect of NCMR implementation on regional medical resources, characterized by *Doctor*, *Nurse,* and *Sickbed*. In columns (1) and (2), the regression coefficients of NCMR, the core explanatory variable, are significantly positive at the 5% level, while the coefficient in column (3) is positive but not significant. This suggests that, compared to non-pilot provinces, the number of doctors and nurses increased by 12.6 and 19.8%, respectively, in the pilot provinces.

**Table 2:** Benchmark regression results based on staggered DID model.

Variables	(1)	(2)	(3)	(4)	(5)
	*Doctor*	*Nurse*	*Sickbed*	*Out-patient*	*In-patient*
*NCMR*	0.126^**^ (0.052)	0.198^**^ (0.095)	0.251 (0.178)	−0.023 (0.028)	−0.072^**^ (0.027)
*Control variables*	Yes	Yes	Yes	Yes	Yes
*Regional FE*	Yes	Yes	Yes	Yes	Yes
*Time FE*	Yes	Yes	Yes	Yes	Yes
*N*	496	496	496	496	496
*R*^2^	0.951	0.950	0.937	0.969	0.977

Statistics in parentheses are robust standard errors, clustered by province; ^⁎⁎^ *p* < 0.05. DID, difference-in-differences; *Doctor*, the number of licensed physicians per 1,000 population; *Nurse*, the number of registered nurses per 1,000 population; *Sickbed*, the number of sickbeds in medical institutions per 1,000 population; *Out-patient*, per capita per time medical expense for out-patients; *In-patient*, per capita medical expense for in-patients; *NCMR*, a core explanatory variable means whether to implement national comprehensive medical reform; *FE*, fixed effect; *N*, the number of samples.

The last two columns show the NCMR’s effect on medical expenditures, characterized by *Out-patient* and *In-patient*. The coefficient in column (4) is negative but not significant, while the coefficient in column (5) is significantly negative, indicating that NCMR played a key role in reducing inpatient medical expenses. *Per capita* inpatient medical costs decreased by 7.2% in the pilot provinces. Overall, the results demonstrate some success in improving the accessibility and affordability of medical resources and services.

#### 4.1.2 Parallel trend test

The validity of the Staggered DID model depends on the parallel trends assumption, which posits that the dependent variables should follow the same trend in both the pilot and non-pilot provinces before policy implementation. To assess this, following the approach of Jacobson et al. (33), we employed the Event Study Method by constructing the following model to investigate parallel trends:


(4) $$Y_{\mathrm{it}}=\alpha +\overset{t=6}{\underset{t=-9}{\sum }}\beta_{t}\times {\mathrm{NCMR}}_{\mathrm{it}}+\gamma X_{\mathrm{it}}+\delta_{t}+\mu_{i}+\varepsilon_{\mathrm{it}}$$

**Figure 2:**
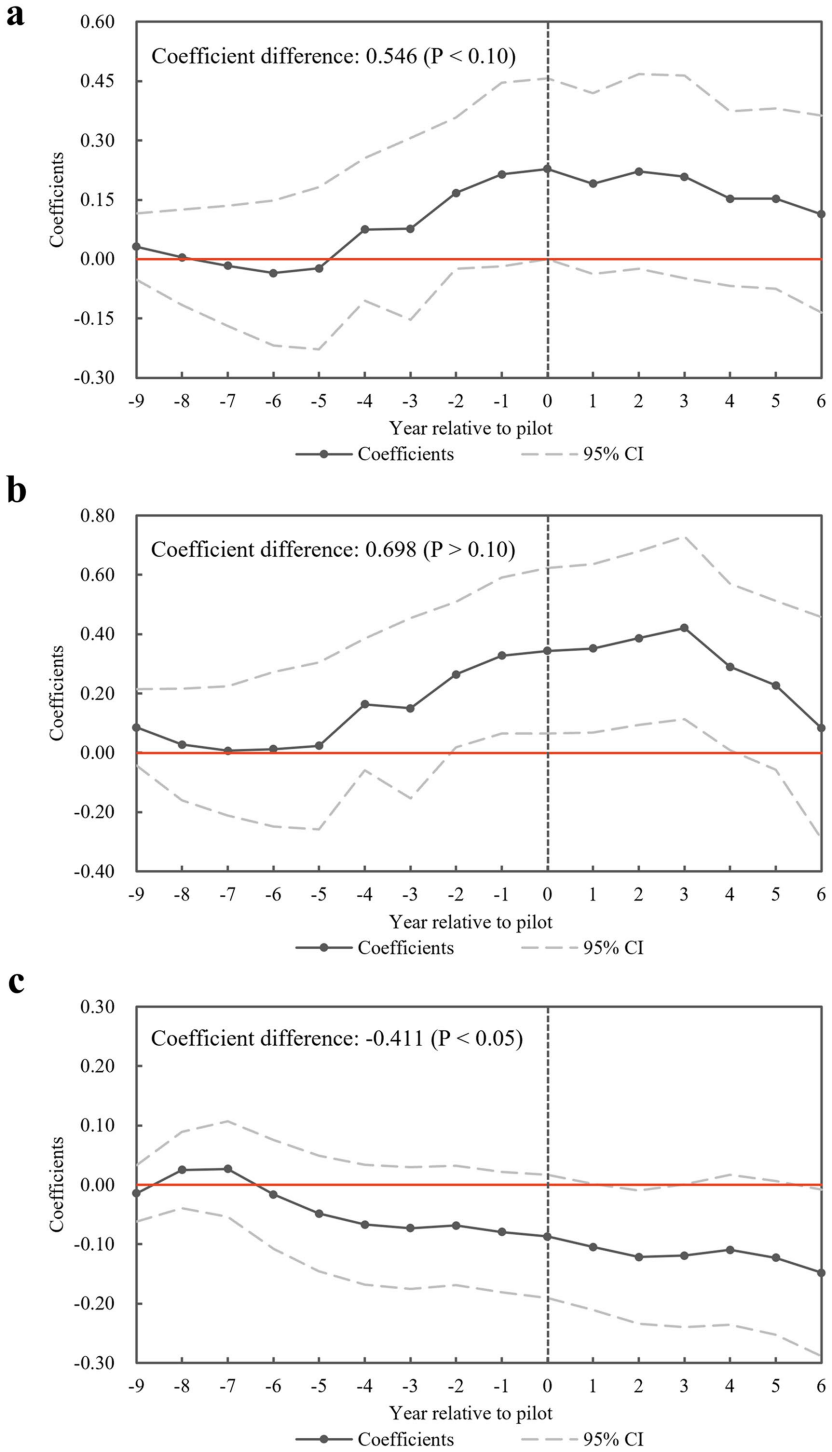
Results of the parallel trend test. **(a)** Test results for dependent variable *Doctor*. **(b)** Test results for dependent variable *Nurse*. **(c)** Test results for dependent variable *In-patient*.

Our focus is on the significance of $\beta_{t}$ , with the criterion for passing the test being a non-significant $\beta_{t}$ prior to the implementation of NCMR ( t<0 ) (Equation 4). The base year is set as 2006 to avoid multicollinearity. The parallel trend test results for *Doctor*, *Nurse*, and *In-Patient* are presented in Figure 2. The non-significant $\beta_{t}$ ( t<0 ) in Figures 2A,C indicates no systematic difference in the number of licensed physicians or inpatient medical expenses between the experimental and control groups before NCMR implementation, confirming the validity of the Staggered DID model.

Additionally, for each dependent variable, we tested the difference in $\beta_{t}$ before and after the policy implementation. The coefficient differences for *Doctor* and *In-Patient* are 0.546 (*p* < 0.10) and-0.411 (*p* < 0.05), respectively, further confirming the impact of NCMR. These results indicate a significant increase in licensed physicians and a notable reduction in inpatient medical expenses following the policy. Next, we will conduct a placebo test for the *Doctor* and *In-patient* variables, which passed the parallel trend test.

#### 4.1.3 Placebo test

**Figure 3:**
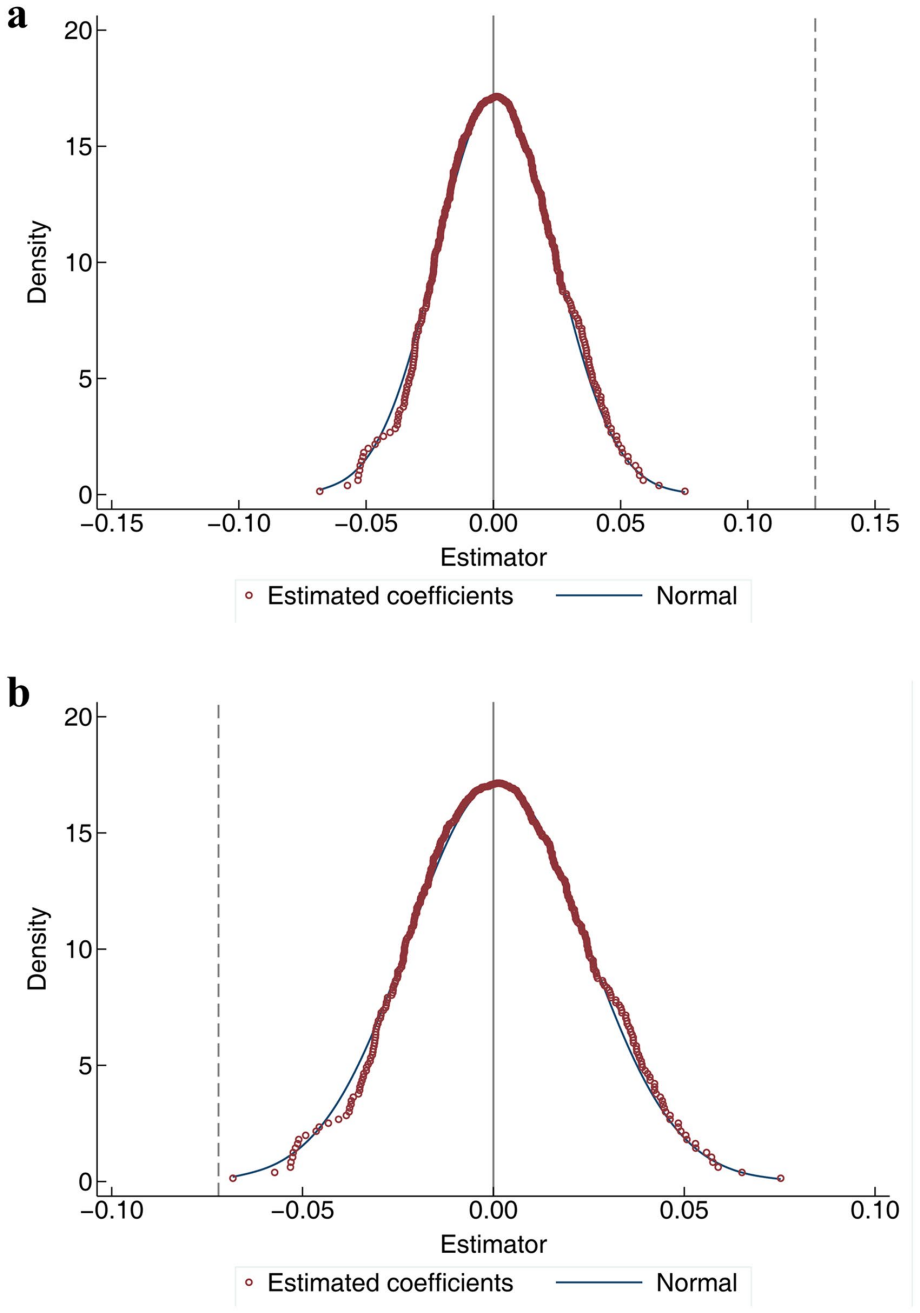
Results of the placebo test. **(a)** Test results for dependent variable *Doctor*. **(b)** Test results for dependent variable *In-patient*.

Admittedly, significant estimates could also result from unobservable random factors. Therefore, the baseline regression results need to be validated by randomly assigning pilot provinces and policy implementation times. Specifically, a new variable $NCMR_{it}^{'}$ is randomly generated to replace the original explanatory variable $NCMR_{it}$ in the regression analysis, producing an incorrect estimation coefficient $\beta^{\prime }$ . This process is repeated 500 times to complete the placebo test (Figure 3).

The placebo tests for *Doctor* and *In-patient* are illustrated in Figures 3A,B. The results show that $\beta^{\prime }$ values are concentrated around 0 and follow a normal distribution, while the true estimation coefficient β (represented by the dashed line) lies outside the range of all the incorrect coefficients. This suggests that the actual estimation is unlikely to have been influenced by random factors, confirming that the placebo test is successfully passed (Figures 3A,B).

### 4.2 Results of SCM test

#### 4.2.1 Provincial policy effect evaluation

**Figure 4:**
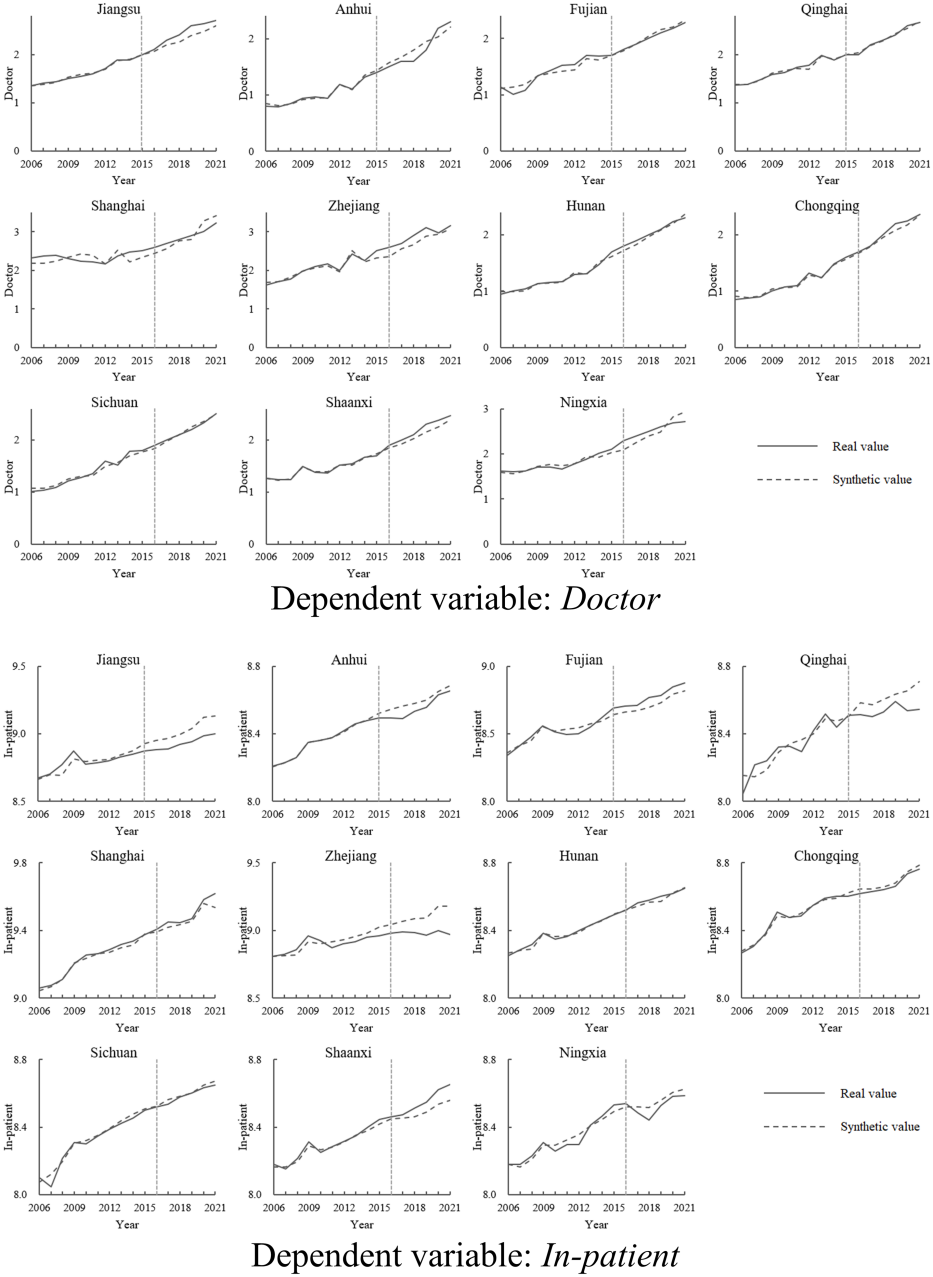
Comparison of real and synthetic values of dependent variables in pilot provinces.

The heterogeneity of policy shocks across different pilot provinces will be examined in this section. Specifically, the impact of NCMR on increasing licensed doctors and reducing in-patient medical expenses in individual pilot provinces will be analyzed using SCM. Table 3 lists the control variables and their weights used by SCM to construct the “synthetic provinces” for the two dependent variables, *Doctor* and *In-patient*. The comparison between each pilot province and its “synthetic province” in terms of *Doctor* and *In-patient* is depicted in Figure 4, with the vertical dashed line representing the implementation of NCMR (2015 or 2016). The closer the real values are to the fitted values before the implementation (left side of the dashed line), the better the fit of the SCM. In contrast, the gap between the real and fitted values after the implementation (right side of the dashed line) reflects the policy effect.

**Table 3:** Weighting combinations used to synthesize pilot provinces.

DV: *Doctor*	Jiangsu	Anhui	Fujian	Qinghai	Shanghai	Zhejiang	Hunan	Chongqing	Sichuan	Shaanxi	Ningxia
Beijing	0	0	0	0.028	0.144	0.140	0.012	0	0	0	0
Tianjin	0	0	0	0	0	0	0	0	0	0	0
Hebei	0	0	0	0	0	0	0	0	0	0	0
Shanxi	0	0	0	0.133	0	0	0	0	0	0	0
Inner Mongolia	0	0	0	0	0	0	0	0	0	0.210	0
Liaoning	0.148	0	0	0	0	0	0	0	0	0	0
Jilin	0	0	0	0	0.856	0	0	0	0	0	0.533
Heilongjiang	0	0	0	0	0	0	0	0	0	0	0
Jiangxi	0	0.166	0	0	0	0	0	0	0	0.090	0
Shandong	0.358	0	0.199	0.451	0	0.860	0	0.142	0.460	0	0.372
Henan	0	0.322	0.401	0	0	0	0.605	0.653	0.285	0.178	0
Hubei	0.379	0	0	0	0	0	0.231	0	0	0	0
Guangdong	0.116	0	0.399	0.107	0	0	0	0	0	0	0
Guangxi	0	0	0	0	0	0	0	0	0.255	0	0
Hainan	0	0	0	0.281	0	0	0.059	0	0	0	0
Guizhou	0	0.512	0	0	0	0	0.066	0.205	0	0.232	0
Yunnan	0	0	0	0	0	0	0	0	0	0	0
Tibet	0	0	0	0	0	0	0.026	0	0	0.042	0.095
Gansu	0	0	0	0	0	0	0	0	0	0	0
Xinjiang	0	0	0	0	0	0	0	0	0	0.248	0

DV: *In-patient*	Jiangsu	Anhui	Fujian	Qinghai	Shanghai	Zhejiang	Hunan	Chongqing	Sichuan	Shaanxi	Ningxia
Beijing	0.119	0	0	0	0.445	0.443	0	0	0.035	0.021	0
Tianjin	0	0.013	0	0	0.416	0	0	0	0	0	0
Hebei	0	0	0	0	0	0	0	0	0	0	0
Shanxi	0	0.020	0	0	0	0	0	0	0	0	0
Inner Mongolia	0	0	0	0	0	0	0	0.078	0.089	0	0
Liaoning	0.374	0	0.536	0	0	0	0	0.301	0.042	0.106	0
Jilin	0	0	0	0	0	0	0	0	0	0	0
Heilongjiang	0	0.075	0	0	0	0	0	0	0	0	0
Jiangxi	0	0	0	0	0	0	0	0	0	0.106	0
Shandong	0	0.121	0	0	0.109	0	0	0	0.057	0.125	0.488
Henan	0	0.139	0	0	0	0	0.090	0	0.495	0	0
Hubei	0	0	0	0	0	0	0	0	0	0	0
Guangdong	0.507	0.186	0.172	0	0.029	0.212	0.320	0	0	0	0
Guangxi	0	0	0	0	0	0	0	0	0	0	0
Hainan	0	0	0	0.549	0	0	0	0.398	0	0	0
Guizhou	0	0	0	0.249	0	0	0	0	0	0.318	0.342
Yunnan	0	0.364	0.293	0	0	0.346	0.590	0.223	0.283	0.323	0.170
Tibet	0	0	0	0.202	0	0	0	0	0	0	0
Gansu	0	0.082	0	0	0	0	0	0	0	0	0
Xinjiang	0	0	0	0	0	0	0	0	0	0	0

DV, dependent variable; *Doctor*, the number of licensed physicians per 1,000 population; *In-patient*, per capita medical expense for in-patients.

From the results, although the average effect of NCMR has been validated by multiple tests, only Jiangsu, Zhejiang, and Chongqing have shown both an increase in the number of licensed physicians and a decrease in in-patient medical expenses following the policy. Additionally, in-patient medical expenses have declined in Anhui, Qinghai, Sichuan, and Ningxia, while Shaanxi has experienced an increase in the number of licensed physicians. However, no notable changes in either variable were observed in Fujian, Shanghai, or Hunan.

#### 4.2.2 Placebo test

In accordance with the permutation test proposed by Abadie et al. (32), a placebo test was conducted for the analyses of *Doctor* and *In-patient* in Jiangsu, Zhejiang, and Chongqing. This test assumed that all non-pilot provinces implemented NCMR in 2015 or 2016, allowing for a comparison of the policy effect between the pilot and non-pilot groups. To enhance accuracy, non-pilot provinces with a root mean square prediction error that was 1.5 times higher than that of the pilot provinces before NCMR implementation were excluded from the analysis.

**Figure 5:**
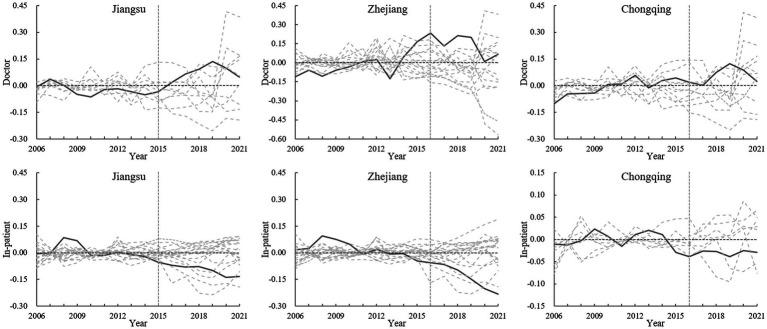
Results of the permutation test.

Figure 5 illustrates the experimental effects in Jiangsu, Zhejiang, and Chongqing (solid lines) compared to those in other non-pilot provinces (dashed lines). For the variable *Doctor*, the effects in the three pilot provinces remain around zero before NCMR implementation (to the left of the vertical dashed line), but increase afterward (to the right of the vertical dashed line). Specifically, the solid lines are consistently above the dashed lines prior to 2019. However, after 2019, the effects in the pilot provinces start to diminish and are gradually surpassed by the placebo effects of several non-pilot provinces, indicating a slight decline in the impact of NCMR on the number of physicians.

In contrast, for the variable *In-patient*, the experimental effects in the three pilot provinces remain substantial after the policy implementation. The solid lines are generally below the majority of the dashed lines, highlighting that NCMR significantly contributes to reducing in-patient medical expenses.

### 4.3 Analysis of policy characteristics

From the perspective of the NCMR policy effect in each pilot province, only Jiangsu, Zhejiang, and Chongqing have successfully achieved both an increase in medical resources and a reduction in residents’ medical burden. This outcome can be attributed to the nature of NCMR as a top-down policy, where each pilot province is required to adapt and tailor the policy details to its specific conditions. Consequently, different pilot provinces may be at various stages of reform and exhibit diverse outcomes. To understand this variation, it is essential to examine the policy characteristics and implementation strategies of Jiangsu, Zhejiang, and Chongqing.

To address this question, we analyzed related policy descriptions and news reports from the official websites of the provincial governments of Jiangsu, Zhejiang, and Chongqing, as well as from mainstream media. We used Python to perform word frequency statistics to examine the specific policy measures associated with NCMR in these provinces. This analysis aims to explore the reasons behind the successful reform outcomes and provide guidance for other provinces implementing healthcare reforms. The word frequency statistics are presented in Table 4. To clarify the differences and focal points in the NCMR policies of these three provinces, we refined the initial statistical results by removing common nouns such as “service,” “medical,” and “health” that appeared in all relevant texts. These terms were excluded as they did not provide insights into specific measures or methods outlined in the policy documents.

**Table 4:** Policy word frequency statistics for Jiangsu, Zhejiang, and Chongqing.

Jiangsu		Zhejiang		Chongqing	
Word	Frequency	Word	Frequency	Word	Frequency
Remuneration	56	Internet	59	Medicine	65
Contract	40	Wisdom	34	Grass-roots level	63
Personnel	34	Information	29	Supervision	51
Expenses	33	Data	26	Purchase	47
Talents	29	Intelligence	25	Information	41
Family doctor	28	Medical community	23	Traceable	32
Salary	25	Digital	22	Flow	21
Medical staff	18	Sinking	20	Pharmaceuticals and equipment	21
Price	17	Sharing	19	Pharmaceutical exchange	16
Subsidy	15	Artificial intelligence	11	Rural towns	10

First, high-frequency words in the policy and news texts from Jiangsu include “remuneration,” “talents,” “salary,” and “medical staff.” As a pioneering economic region and one of the initial NCMR pilot areas, Jiangsu started with a strong foundation and considerable momentum for reform. The focus here has been on improving the remuneration of medical personnel and addressing medical expenses, with a variety of supporting policies introduced. Specifically, to increase the number of medical staff and enhance their motivation, Jiangsu has implemented salary reforms, such as encouraging an annual salary system for hospital leaders and offering annual or negotiated salaries for high-level talents. Additionally, efforts to reduce medical expenses have included price reforms to lower the proportion of drug expenditures for both out-patient and in-patient treatments (34). Control indicators have also been established to monitor and manage the growth of medical expenses.

Second, high-frequency words in Zhejiang’s policy and news texts include “Internet,” “data,” “medical community,” and “sinking.” Zhejiang’s approach to NCMR emphasizes “digital health” and the development of “Internet and medical health” services. The province has focused on AI-assisted clinical decision support, electronic medical insurance billing via blockchain, and mobile payment systems to enhance healthcare delivery and data sharing. Unified platforms for electronic prescriptions and Internet hospital services are being developed to ensure data traceability, whole-process supervision, and more efficient diagnosis and treatment. Additionally, Zhejiang has completed the construction, evaluation, and monitoring of county-level medical communities to promote the creation of digital medical communities and facilitate the “double-sinking” of medical resources (both hospitals and doctors), as well as improve management, financing, and investment (35). This approach has led to significant improvements in the medical capacity and environment of primary hospitals, enhancing the accessibility of health resources (36).

Third, Chongqing, located in relatively underdeveloped western China, provides a replicable model for the second batch of pilot provinces. High-frequency words in the policy and news texts include “medicine,” “supervision,” “traceable,” and “pharmaceutical exchange” (Table 4). To reduce residents’ medical burden, Chongqing has established a traceability system for medical information, ensuring full-process traceability of pharmaceuticals and equipment from “manufacturers-operators-institutions-patients.” This system enhances drug supply reliability and has led to a 53% reduction in the prices of 157 drugs. For improving resource accessibility, Chongqing integrates urban and rural medical resources by supporting collaborations between municipal hospitals and district health centers, and promoting the development of multi-district hospitals. Additionally, Chongqing guides medical personnel to grassroots levels and fosters the co-construction and sharing of medical resources, which is likely to enhance individual satisfaction with medical care.

### 4.4 Extensibility analysis

#### 4.4.1 Factors influencing policy effectiveness

The results of the basic regression analysis indicated that NCMR generally increased medical resources, as measured by the number of licensed physicians (*Doctor*), and alleviated the medical burden of residents, as reflected by in-patient medical expenditure (*In-patient*) in the pilot provinces. However, the policy effects varied across different provinces. What factors might influence the effectiveness of NCMR? To address this question, this study will explore several potential influencing factors.

1. The degree of population aging, measured by the proportion of individuals aged over 65 in the total population. Older adult individuals typically have a *per capita* out-patient volume and hospitalization rate 3–4 times higher than those of children, youth, and adults (37), leading to greater demand for medical resources. Additionally, a higher proportion of older adult people is a significant factor contributing to increased healthcare expenditure (38). Therefore, the degree of population aging is expected to influence the policy effectiveness of NCMR. Provinces with a lower degree of population aging may achieve better reform results.
2. Population health status, which is often measured by mortality rates (39, 40). In this study, mortality is used as an indicator of regional population health status, with lower mortality reflecting higher overall health levels. Generally, poorer population health conditions lead to greater demand for medical resources and a heavier medical burden. Therefore, NCMR is expected to achieve better outcomes in provinces with better population health status.

#### 4.4.2 Extensibility analysis results

Before analysis, the samples were grouped based on the upper and lower tertiles of the proportion of the older adult population and regional mortality rates, respectively. Samples above the upper tertile were categorized as the “high degree of population aging (high-AG)/low population health status (low-HE)” group, while those below the lower tertile were classified as the “low degree of population aging (low-AG)/high population health status (high-HE)” group. The resulting sub-samples were then analyzed using the Staggered DID model, and the results are presented in Table 5.

**Table 5:** Extensibility analysis results.

Model	(1)	(2)	(3)	(4)	(5)	(6)	(7)	(8)
Basis for grouping	High-AG	Low-AG	High-AG	Low-AG	High-HE	Low-HE	High-HE	Low-HE
Variables	*Doctor*		*In-patient*		*Doctor*		*In-patient*	
NCMR	0.100^**^ (0.046)	0.199^**^ (0.078)	−0.007 (0.017)	−0.100^*^ (0.048)	0.405^***^ (0.068)	0.013 (0.033)	−0.073 (0.042)	−0.008 (0.020)
Control variables	Yes	Yes	Yes	Yes	Yes	Yes	Yes	Yes
Regional FE	Yes	Yes	Yes	Yes	Yes	Yes	Yes	Yes
Time FE	Yes	Yes	Yes	Yes	Yes	Yes	Yes	Yes
N	147	146	147	146	150	147	150	147
*R*^2^	0.980	0.933	0.994	0.972	0.950	0.988	0.991	0.981

Statistics in parentheses are robust standard errors, clustered by province; ^⁎^ *p* < 0.10, ^⁎⁎^ *p* < 0.05, ^⁎⁎⁎^ *p* < 0.01.

From columns (1) and (2), as well as (3) and (4), it is evident that the low-AG group has experienced more pronounced policy effects following the implementation of NCMR compared to the high-AG group. Specifically, the low-AG group shows higher estimates for increasing the number of licensed physicians (0.199** vs. 0.100**) and more significant reductions in in-patient medical expenses (−0.100* vs. −0.007).

Similarly, columns (5) and (6) reveal that the high-HE group experienced a greater policy effect in increasing the number of licensed physicians. However, in columns (7) and (8), despite non-significant coefficients due to the reduced sample size, the high-HE group achieved a lower reduction in in-patient medical expenses compared to the low-HE group, with a significant difference (−0.066, *p* < 0.05) between the two groups. This indicates that the high-HE group performed better in reducing in-patient medical expenses to a certain extent.

On one hand, considering the relationship between population aging and medical resources, the increasing older adult population has significantly expanded the demand for medical resources. However, the current adjustments to medical resources in China have not fully kept pace with the growth in the older adult population, leading to an overall insufficiency in medical resources (41). Additionally, the rise in the older adult population accelerates the depletion of available medical resources (42). Consequently, population aging negatively impacts the availability of medical resources and poses a challenge to the effectiveness of the NCMR policy.

On the other hand, population aging increases the depreciation rate of health capital, with older individuals requiring more investment in healthcare services (43). This, in turn, amplifies the impact on public health expenditure (44). Many studies have identified aging as a significant driver of rising healthcare spending, projecting that by 2025, approximately 20% of the increase in healthcare costs will be attributed to an aging population (45, 46). Although provinces with higher levels of aging have substantial potential for reform, the dual challenge of increased demand for medical resources and elevated healthcare expenditure due to aging can create greater resistance to reform. This resistance can lead to less effective outcomes for the NCMR in regions with higher population aging.

Additionally, the impact of regional overall population health status on the effectiveness of NCMR reveals that the policy is less effective in areas with poorer health outcomes. Consistent with our findings, Sun et al. (47) observed that medical resources and health conditions in rural areas of China lagged significantly behind those in urban areas. This disparity poses a substantial challenge for healthcare reform, resulting in notably weaker effects of NCMR in rural regions compared to urban areas with better health levels. Poorer health conditions typically lead to higher demand for medical services and increased medical expenses, creating additional obstacles for NCMR in improving medical resources and reducing costs.

## 5 Discussion

Enhancing the accessibility of medical resources and establishing an affordable medical care system for residents have long been primary goals of global healthcare reform. This study effectively estimates and verifies the average impact of China’s NCMR on increasing the number of licensed physicians and reducing in-patient medical expenses using the staggered DID model and a series of tests. Additionally, SCM results highlight that NCMR achieves these effects only in certain pilot provinces, with noticeable provincial heterogeneity influenced by factors such as population aging and health status. These findings offer valuable macro-level empirical evidence and can guide adjustments to the reform strategy in the next stage.

Our study shows that, in the pilot provinces, NCMR effectively increases the number of licensed physicians but has no significant impact on the number of registered nurses and sickbeds in healthcare institutions. This may be because NCMR primarily focused on enhancing the training and number of general practitioners. By implementing license registration reforms, expanding the scope of practice, ensuring adequate remuneration, and prioritizing general practitioners, the policy has led to a noticeable increase in the number of practicing physicians. However, this also underscores the need for future expansion in other medical resources, such as nurses and sickbeds.

Regarding the affordability of medical services, NCMR effectively reduces *per capita* medical expenses for in-patients but has no impact on *per capita* out-patient medical expenses. This aligns with findings from Wang et al. (48) and Nie et al. (49). The likely reason is that NCMR, as a comprehensive policy, aims to balance reducing the medical burden on residents while maintaining the financial sustainability of medical staff and hospital operations. Specifically, by canceling drug markups and reducing inflated drug prices, NCMR shifts some financial burden from pharmaceuticals to medical services. This shift is intended to reflect the labor value of medical personnel and adjust hospital income structures, reducing reliance on pharmacy profits to subsidize medical services. Consequently, while drug expenses decrease, the overall out-patient expenditure—which includes drug costs, medical examinations, and service fees—remains unchanged (48).

Regarding provincial heterogeneity, Jiangsu, Zhejiang, and Chongqing have successfully achieved both an increase in medical resources, as indicated by the number of physicians, and a reduction in residents’ medical burden, as measured by in-patient expenses. In contrast, Anhui, Qinghai, Sichuan, and Ningxia have only managed to reduce in-patient expenses, while Shaanxi has only succeeded in increasing the number of qualified doctors. The remaining provinces have not shown the expected improvements. This highlights that, within a top-down reform framework, each pilot province must tailor its implementation and detailed reform regulations to its local conditions and existing medical system infrastructure. As a result, the stages and outcomes of the reform vary across different provinces.

However, there are some limitations to this study. First, our evaluation of the NCMR policy effects is conducted from a macro-level perspective (provincial level), and lacks analysis at the meso-level (e.g., public hospitals) and micro-level (e.g., individual residents). Second, the study relies on traditional causal inference models for evaluation. Future research could benefit from incorporating big data and AI technologies, utilizing machine learning models to more accurately identify and quantify policy effects.

## 6 Conclusion

Based on provincial panel data from mainland China spanning 2006–2021, this study employs the Staggered DID model, SCM, and grouping regression to evaluate the average policy effect of NCMR and explore provincial heterogeneity and influencing factors. The findings are as follows: First, with regard to the accessibility of medical resources, NCMR effectively increased the number of licensed physicians by 12.6% in the pilot provinces. Second, with regard to the affordability of medical services, NCMR reduced the *per capita* medical expense for in-patients by 7.2%. Third, the policy effects varied across provinces. Jiangsu, Zhejiang, and Chongqing achieved both an increase in the number of licensed physicians and a decrease in in-patient medical expenses, whereas other provinces either saw only one of these effects or no significant changes. Finally, the effectiveness of NCMR is influenced by population aging and health status. Provinces with a low-AG and high-HE experienced more pronounced reform effects.

## Data Availability

The original contributions presented in the study are included in the article/supplementary material, further inquiries can be directed to the corresponding author.

## References

[1] Porter ME Teisberg EO. Redefining competition in health care. Harv Bus Rev. (2004) 82:64–76, 136. 15202288

[2] Du C Zhu HP. The evolutionary logic of urban health Care Systems in China. Soc Sci China. (2016) 08:66–89+205–6.

[3] Grossman SJ Hart OD. The costs and benefits of ownership-a THEORY of vertical and lateral integration. J Polit Econ. (1986) 94:691–719. doi: 10.1086/261404

[4] Li H Dong SP Liu TF. Relative efficiency and productivity: a preliminary exploration of public hospitals in Beijing, China. BMC Health Serv Res. (2014) 14:14. doi: 10.1186/1472-6963-14-158, PMID: 24708701 PMC3986456

[5] Liu G Krumholz S. Economics of health transitions in China. The Oxford companion to the economics of China (2014).

[6] Yip WN Fu HQ Chen AT Zhai TM Jian WY Xu RM. 10 years of health-care reform in China: progress and gaps in universal health coverage. Lancet. (2019) 394:1192–204. doi: 10.1016/S0140-6736(19)32136-1 31571602

[7] Liu GG Vortherms SA Hong XZ. China's health reform update. Annu Rev Publ Health. (2017) 38:431–48. doi: 10.1146/annurev-publhealth-031816-044247 28125384

[8] Dong ZY Zhao CX. “A new round of medical and health system reform”: achievements, difficulties and path selection, Reform. (2020) 09:149–59.

[9] Zhang XP Xiong YQ Ye J Deng ZH Zhang XP. Analysis of government investment in primary healthcare institutions to promote equity during the three-year health reform program in China. BMC Health Serv Res. (2013) 13:13. doi: 10.1186/1472-6963-13-114, PMID: 23530658 PMC3614483

[10] Meng QY Yin DX Mills A Abbasi K. China's encouraging commitment to health. BMJ. (2019) 365:2. doi: 10.1136/bmj.l4178, PMID: 31227519 PMC6598728

[11] Wan SD Chen YM Xiao YJ Zhao QQ Li MC Wu SQ. Spatial analysis and evaluation of medical resource allocation in China based on geographic big data. BMC Health Serv Res. (2021) 21:1084. doi: 10.1186/s12913-021-07119-3, PMID: 34641850 PMC8508408

[12] Yuan L Cao J Wang D Yu D Liu G Qian ZX. Regional disparities and influencing factors of high quality medical resources distribution in China. Int J Equity Health. (2023) 22:8. doi: 10.1186/s12939-023-01825-6, PMID: 36627636 PMC9832614

[13] Lu C Zhang ZX Lan XT. Impact of China's referral reform on the equity and spatial accessibility of healthcare resources: a case study of Beijing. Soc Sci Med. (2019) 235:112386. doi: 10.1016/j.socscimed.2019.112386, PMID: 31272079

[14] Hao Y Liu S Lu ZN Huang JB Zhao MY. The impact of environmental pollution on public health expenditure: dynamic panel analysis based on Chinese provincial data. Environ Sci Pollut R. (2018) 25:18853–65. doi: 10.1007/s11356-018-2095-y, PMID: 29713982

[15] Li H Lu J Li B. Does pollution-intensive industrial agglomeration increase residents' health expenditure? Sustain Cities Soc. (2020) 56:102092. doi: 10.1016/j.scs.2020.102092

[16] Han HC Hai CX Wu TQ Zhou NC. How does digital infrastructure affect residents' healthcare expenditures? Evidence from Chinese microdata. Front. Public Health. (2023) 11:11. doi: 10.3389/fpubh.2023.1122718, PMID: 37213630 PMC10192711

[17] Baji P Pavlova M Gulácsi L Groot W. Changes in equity in out-of-pocket payments during the period of health care reforms: evidence from Hungary. Int J Equity Health. (2012) 11:36. doi: 10.1186/1475-9276-11-36, PMID: 22828250 PMC3439316

[18] Limwattananon S Neelsen S O'Donnell O Prakongsai P Tangcharoensathien V van Doorslaer E. Universal coverage with supply-side reform: the impact on medical expenditure risk and utilization in Thailand. J Public Econ. (2015) 121:79–94. doi: 10.1016/j.jpubeco.2014.11.012

[19] Zhu DW Shi XF Chen SY Ye X He P. Impacts of price changes on public hospital reforms in China: evidence from 25 million patients at tertiary hospitals. Health Policy Plan. (2022) 37:1307–16. doi: 10.1093/heapol/czac073, PMID: 36057091

[20] Liu LM Xu Y Yu JF Man XW Jiang Y Zhao LY. The impact of comprehensive public hospital reforms on the direct medical cost of inpatients with coronary heart disease. Front. Public Health. (2022) 10:10. doi: 10.3389/fpubh.2022.891186, PMID: 36159309 PMC9500355

[21] Liu X Xu J Yuan B Ma X Fang H Meng Q. Containing medical expenditure: lessons from reform of Beijing public hospitals. BMJ. (2019) 365:l2369. doi: 10.1136/bmj.l2369, PMID: 31227508 PMC6598718

[22] Gao L Shi LY Meng QY Kong XR Guo MN Lu F. Effect of healthcare system reforms on public hospitals' revenue structures: evidence from Beijing, China. Soc Sci Med. (2021) 283:114210. doi: 10.1016/j.socscimed.2021.114210 34274783

[23] Liu WL Xia Y Hou JL. Health expenditure efficiency in rural China using the super-SBM model and the Malmquist productivity index. Int J Equity Health. (2019) 18:111. doi: 10.1186/s12939-019-1003-5, PMID: 31324184 PMC6642491

[24] Sun XY Xu L Adnan KMM Luo YS. Can comprehensive medical reform improve the efficiency of medical resource allocation? Evidence from China. Int J Public Health. (2023) 68:68. doi: 10.3389/ijph.2023.1606602, PMID: 38179320 PMC10764414

[25] Sheng WX Wan L Wang CY. The spillover effect of fiscal environmental protection spending on residents' medical and healthcare expenditure: evidence from China. Environ Geochem Health. (2022) 44:2975–86. doi: 10.1007/s10653-021-01146-z, PMID: 34762256

[26] Liu H. Health depreciation effect and medical cost effect of air pollution: based on multidimensional health perspective. Air Qual Atmos Health. (2022) 15:877–92. doi: 10.1007/s11869-022-01189-w

[27] Tseng CH Lei C Chen YC. Evaluating the health costs of oral hexavalent chromium exposure from water pollution: a case study in Taiwan. J Clean Prod. (2018) 172:819–26. doi: 10.1016/j.jclepro.2017.10.177

[28] Xu WZ Lin J. Fiscal decentralization, public health expenditure and public health-evidence from China. Front public. Health. (2022) 10:10. doi: 10.3389/fpubh.2022.773728, PMID: 35664120 PMC9157548

[29] Beck T Levine R Levkov A. Big bad banks? The winners and Losers from Bank deregulation in the United States. J Financ. (2010) 65:1637–67. doi: 10.1111/j.1540-6261.2010.01589.x

[30] Abadie A Gardeazabal J. The economic costs of conflict: a case study of the Basque Country. Am Econ Rev. (2003) 93:113–32. doi: 10.1257/000282803321455188

[31] Chen F Chen Q Hou J Li S. Effects of China's carbon generalized system of preferences on low-carbon action: a synthetic control analysis based on text mining. Energy Econ. (2023) 124:106867. doi: 10.1016/j.eneco.2023.106867

[32] Abadie A Diamond A Hainmueller J. Synthetic control methods for comparative case studies: estimating the effect of California's tobacco control program. J Am Stat Assoc. (2010) 105:493–505. doi: 10.1198/jasa.2009.ap08746

[33] Jacobson LS Lalonde RJ Sullivan DG. Earnings losses of displaced workers. Am Econ Rev. (1993) 83:685–709.

[34] Zang X Zhang MR Wei SH Tang WX Jiang S. Impact of public hospital pricing reform on medical expenditure structure in Jiangsu, China: a synthetic control analysis. BMC Health Serv Res. (2019) 19:512. doi: 10.1186/s12913-019-4357-x, PMID: 31337396 PMC6651988

[35] Wu C Tu YX Li ZX Yu JX. An early assessment of the county medical community reform in China: a case study of Zhejiang province. J Chin Gov. (2021) 6:463–85. doi: 10.1080/23812346.2021.1978722

[36] Sun ZS Wang SH Zhao HJ Zhou X Zhang LD Shi JP. Does descending health resources reform impact patient low-level hospital selection behavior? Evidence from Zhejiang, China. Arch Public Health. (2021) 79:179. doi: 10.1186/s13690-021-00700-6, PMID: 34663478 PMC8522119

[37] Wang CY Li F Wang LN Zhou WT Zhu BF Zhang XX. The impact of population aging on medical expenses: a big data study based on the life table. Biosci Trends. (2017) 11:619–31. doi: 10.5582/bst.2017.01243, PMID: 29225282

[38] Lopreite M Mauro M. The effects of population ageing on health care expenditure: a Bayesian VAR analysis using data from Italy. Health Policy. (2017) 121:663–74. doi: 10.1016/j.healthpol.2017.03.015, PMID: 28392027

[39] Gennaro V Cervellera S Cusatelli C Miani A Pesce F De Gennaro G. Use of official municipal demographics for the estimation of mortality in cities suffering from heavy environmental pollution: results of the first study on all the neighborhoods of Taranto from 2011 to 2020. Environ Res. (2022) 204:112007. doi: 10.1016/j.envres.2021.112007, PMID: 34509482

[40] Antonelli MA Marini G. Do institutions matter for citizens' health status? Empirical evidence from Italy. Eur J Health Econ. (2024):21. doi: 10.1007/s10198-024-01689-9, PMID: 38722437 PMC11743420

[41] Ma M Shi L Xie W Zhu Q Luo J Liao S. Coupling coordination degree of healthcare resource supply, demand and elderly population change in China. Int J Equity Health. (2024) 23:147. doi: 10.1186/s12939-024-02236-x, PMID: 39049064 PMC11270932

[42] Barer ML Evans RG Hertzman C Lomas J. Aging and health care utilization: new evidence on old fallacies. Soc Sci Med. (1987) 24:851–62. doi: 10.1016/0277-9536(87)90186-9, PMID: 3616679

[43] Grossman M. Concept of health capital and demand for health. J Polit Econ. (1972) 80:223–55. doi: 10.1086/259880

[44] Howdon D Rice N. Health care expenditures, age, proximity to death and morbidity: implications for an ageing population. J Health Econ. (2018) 57:60–74. doi: 10.1016/j.jhealeco.2017.11.001, PMID: 29182935

[45] Dieleman JL Squires E Bui AL Campbell M Chapin A Hamavid H. Factors associated with increases in US health care spending, 1996-2013. JAMA. (2017) 318:1668–78. doi: 10.1001/jama.2017.15927, PMID: 29114831 PMC5818797

[46] Keehan SP Stone DA Poisal JA Cuckler GA Sisko AM Smith SD. National Health Expenditure Projections, 2016-25: Price increases, aging push sector to 20 percent of economy. Health Aff (Millwood). (2017) 36:553–63. doi: 10.1377/hlthaff.2016.1627, PMID: 28202501

[47] Sun YP Zhang ZY Sun GY. Health and medical service effects of China’s health care system reform: evidence from “comprehensive medical reform”, Systems Engineering-Theory & Practice (2024).

[48] Wang CC Zha ZY. Has the comprehensive medical reform pilot alleviated the problems of the accessibility and affordability of health care? Public Financ Res. (2021) 12:79–92. doi: 10.19477/j.cnki.11-1077/f.2021.12.005

[49] Nie CF Feng Y. The impact of national comprehensive medical reform on residents' medical expenses: evidence from China. Front Public Health. (2023) 10:15. doi: 10.3389/fpubh.2022.1038543, PMID: 36684899 PMC9850085

